# PSL-LCCL: a resource for subcellular protein localization in liver cancer cell line SK_HEP1

**DOI:** 10.1093/database/baab087

**Published:** 2022-01-17

**Authors:** Fang Huang, Xia Tang, Bo Ye, Songfeng Wu, Keyue Ding

**Affiliations:** Medical Genetic Institute of Henan Province, Henan Provincial People’s Hospital, Henan Key Laboratory of Genetic Disease and Functional Genomics, National Health Commission Key Laboratory of Birth Defect Prevention, Henan Provincial People’s Hospital of Henan University, People’s Hospital of Zhengzhou University, #7 Road Weiwu, Jinshui District, Zhengzhou, Henan 450003, People’s Republic of China; Department of Bioinformatics, School of Basic Medicine, Chongqing Medical University, #1 Road Yixueyuan, Yuzhong District, Chongqing 400016, People’s Republic of China; State Key Laboratory of Proteomics, Beijing Proteome Research Center, National Center for Protein Sciences (Beijing), Research Unit of Proteomics & Research and Development of New Drug of Chinese Academy of Medical Sciences, Institute of Lifeomics, #38 life science park, Changping District, Beijing 102206, People’s Republic of China; Department of Bioinformatics, School of Basic Medicine, Chongqing Medical University, #1 Road Yixueyuan, Yuzhong District, Chongqing 400016, People’s Republic of China; Medical Genetic Institute of Henan Province, Henan Provincial People’s Hospital, Henan Key Laboratory of Genetic Disease and Functional Genomics, National Health Commission Key Laboratory of Birth Defect Prevention, Henan Provincial People’s Hospital of Henan University, People’s Hospital of Zhengzhou University, #7 Road Weiwu, Jinshui District, Zhengzhou, Henan 450003, People’s Republic of China

## Abstract

The characterization of subcellular protein localization provides a basis for further understanding cellular behaviors. A delineation of subcellular localization of proteins on cytosolic membrane-bound organelles in human liver cancer cell lines (hLCCLs) has yet to be performed. To obtain its proteome-wide view, we isolated and enriched six cytosolic membrane-bound organelles in one of the hLCCLs (SK_HEP1) and quantified their proteins using mass spectrometry. The vigorous selection of marker proteins and a machine-learning-based algorithm were implemented to localize proteins at cluster and neighborhood levels. We validated the performance of the proposed method by comparing the predicted subcellular protein localization with publicly available resources. The profiles enabled investigating the correlation of protein domains with their subcellular localization and colocalization of protein complex members. A subcellular proteome database for SK_HEP1, including (i) the subcellular protein localization and (ii) the subcellular locations of protein complex members and their interactions, was constructed. Our research provides resources for further research on hLCCLs proteomics.

**Database URL**: http://www.igenetics.org.cn/project/PSL-LCCL/

## Introduction

The presence of subcellular compartments within eukaryotic cells provides specialized location and physical and chemical environment for protein expression, playing an essential role in cellular homeostasis (1). Furthermore, tight regulation of subcellular protein localization is vital for controlling cell physiology, and their mislocalization has been a critical feature in various cancer cells (2, 3). Thus, the knowledge of the spatial distribution of proteins at the subcellular level is essential for fully understanding cellular behaviors.

Spatial proteomics (4) is an emerging field for mapping all proteins’ locations within the cell that enables a systematic view of subcellular structure (1). Recent developments in proteomics have provided an avenue for detecting thousands of proteins in multiple subcellular compartments simultaneously (5–7). Itzhak *et al.* (6) constructed an organelle map for the HeLa cell by parsing the localization of 8710 proteins and then identifying translocation events after EGF treatment. The temporal and spatial changes in organelle proteome characterized the interaction between the host and virus in human cytomegalovirus-infected fibroblasts (5). An investigation for five cancer cell lines [i.e. A431 (epidermoid carcinoma), U251 (glioblastoma), MCF7 (breast cancer), NCI-H322 and HCC-827 (lung cancer)] revealed that most proteins have a single primary subcellular location, and alternative splicing seldom affects their subcellular localization (8). Davies *et al.* (9) applied the dynamic organellar maps in AP-4-deficient cells to prove that AP-4 vesicles mediate the cellular distribution of the autophagy protein (ATG9A), crucial for autophagosome biogenesis and neuronal maintenance.

 A traditional subcellular fractionation approach to spatial proteomics enriches a particular organelle, followed by mass spectrometry (MS) based protein identification (1). It has been successfully used to define proteomes in individual organelles, including lipid droplets (10), lysosome and transport vesicles (11) and mitochondria (12). However, most organelles are not amenable to genuine ‘purification’. For example, the ‘purified’ mitochondria contained a fraction of endoplasmic reticulum proteins (12). The nucleosol marker PARP could be identified in the cytoplasm membrane-bound organelles (13). Thus, subcellular fractionation may not isolate ‘pure’ fractions of organelles but rather an enrichment (14).

Human liver cancer is the sixth incidence and the third leading cause of cancer-related mortality worldwide (15). The Cancer Cell Line Encyclopedia compiled genetic aberrants and mRNA expression in 25 human liver cancer cell lines (hLCCLs) (16). In addition, the liver hepatocellular carcinoma (LIHC) cohort in TCGA characterized molecular profiles of genomic aberrants, epigenetic and expression signatures (16). Recently, an established Liver cancer cell Model REpository, including 81 hLCCLs, provided a resource for promoting liver cancer drug discovery (17). In addition, a proteomics study on the qualitative and quantitative changes of proteins underlying hepatocarcinogenesis has implications for biomarker screening and therapeutic implications (18). However, the knowledge gap remains that an organelle map for hLCCLs has yet to be portrayed.

Our previous study identified a component of the retromer complex—VPS35—exerting its oncogenic role on LIHC through FGFR3 recycling (19). To further investigate its role in protein sorting and recycling, we characterized the spatial proteome in six cytosolic membrane-bound organelles in SK_HEP1 using a MS-based pipeline to separate and enrich organelles. We clarified the subcellular protein localization in SK_HEP1 and provided a resource for further community use (http://www.igenetics.org.cn/project/PSL-LCCL/).

## Materials and methods

**Figure 1.**
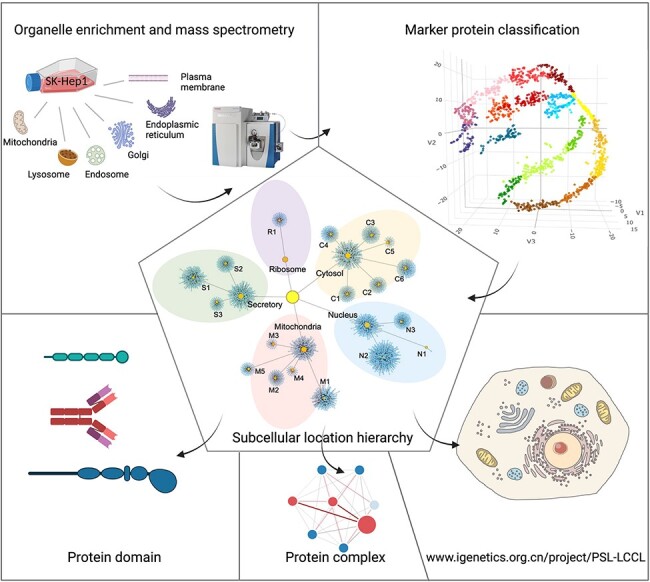
A graphical abstract for the present study. Six cytosolic membrane-bound organelles were isolated and enriched from SK_HEP1, and proteins in each organelle were quantified by MS. A compiled list of marker proteins was clustered and trained using a machine-learning-based algorithm. All proteins were localized at the cluster and neighborhood levels, respectively, as shown in a hierarchical structure. The localization of protein domains and complexes was further investigated. The proteome for subcellular organelles was available at www.igenetics.org.cn/PSL-LCCL.

The framework for the present study included isolation and enrichment of six membrane-bound organelles, MS and data analysis (Figure 1).

### The culture of SK_HEP1 cells

The parental SK_HEP1 was obtained from the Chinese Academy of Sciences (Shanghai, China) and was confirmed free of mycoplasma (MycoAlert PLUS kit; Lonza, Basel, Switzerland). In addition, short tandem repeat profiling confirmed cell authentication (Beijing Microread Gene Technology Co., Beijing, China). We have previously established a VPS35-knockout (KO) model and demonstrated the oncogenic role of VPS35 in the development of liver cancer (19). Both parental and VPS35-KO SK_HEP1 cells were cultured under the same protocol described previously (19). SK_HEP1 cells were passed for 11 generations with an available concentration of $1 \times {10^8}$.

### Protein extraction and digestion

SK_HEP1 cells were mechanically lysed to release organelles. Six targeted membrane-bound organelles, including the plasma membrane, endoplasmic reticulum, endosome, lysosome, Golgi apparatus and mitochondria, were extracted using the Minute organelle Protein Isolation Kit (Invent Biotechnologies Inc., MN), respectively. These organelles were derived from the cultured cells in simultaneous batches in technical triplicate. The organelle was lysed in lysis buffer (8 M urea, 100 mM ammonium bicarbonate and pH 8.0) supplemented with protease inhibitors for 20 min on ice. Samples were then sonicated for 2 min (3 s on and 3 s off) on ice and centrifuged at 14 000 *g* for 10 min. The supernatants were collected, and the protein concentration was measured using Bradford protein assay. Extracted proteins were reduced in 10 mM dithiothreitol at 56℃ for 60 min and then alkylated in 45 mM iodoacetamide at room temperature for 30 min in darkness. The sample was diluted four times by adding 25 mM ammonium bicarbonate buffer, then underwent trypsin digestion (enzyme-to-substrate ratio of 1:50 at 37℃ for 16 h) followed by desalting through C18 cartridges (Beijing Qinglian Biotech, China) and vacuum-dried by Speed Vac. The extracted proteins for each organelle were subjected to Western Blot analysis using organelle-specific marker proteins for confirmation (Supplementary Figure S1A and Supplementary Table S6).

### Mass spectrometry

A 120-min gradient elution separated peptides at a flow rate of 0.300 μL/min with the EASY-nLC 1000 system, directly interfaced with the Thermo Orbitrap Fusion mass spectrometer. The analytical column was purchased from Thermofisher (75 μm ID, 150 mm length; packed with C-18 resin). Mobile phase A consisted of 0.1% formic acid, and mobile phase B consisted of 100% acetonitrile and 0.1% formic acid. The Orbitrap Fusion mass spectrometer was operated in the data-dependent acquisition mode using Xcalibur3.0 software, and there is a single full-scan mass spectrum in the Orbitrap (350–1550 m/z, 120 000 resolution) followed by 3-s data-dependent MS/MS scans in an Ion Routing Multipole at 38% normalized collision energy (HCD). MS was conducted in Beijing Qinglian Biotech (Beijing, China).

### The identification and quantitation of protein

The MS/MS spectra from each LC-MS/MS run were searched against protein sequences from UniProt using Maxqant (Computational Systems Biochemistry under Prof. Jürgen Cox, DEU). The search criteria included that a complete tryptic specificity was required, two missed cleavage was allowed and carbamidomethylation (C) was set as the fixed modifications. The oxidation (M) was set as the variable modification, and precursor ion mass tolerances were set at 15 ppm for all MS acquired in an orbitrap mass analyzer. The fragment ion mass tolerance was set at 20 mmu for all MS2 spectra acquired. The peptide false discovery rate (FDR) was calculated using Target Decoy PSM Validator provided by maxquant. When searched against the reverse decoy database, FDR was determined based on PSMs. Peptides only assigned to a given protein group were considered unique. The FDR was set to 0.01 for protein identifications.

### Marker protein selection

Marker proteins specifically localized in organelles are required to be highly replicable and have robust fractionation profiles under different conditions (8). Therefore, we compiled marker proteins to classify subcellular protein locations using the previously suggested method (8). First, in each organelle, the quantified protein levels were normalized based on the median of each replicate. Second, proteins identified in both parental and VPS35-KO cells were selected. Third, proteins with a Pearson’s correlation coefficient (PCC) $<$0.8 in triplicate were filtered. Finally, proteins with a PCC $<$0.8 or a Spearman correlation coefficient (SCC) $<$0.6 between parental and VPS35-KO cells were filtered (Supplementary Figure S2B). The remaining 1481 proteins were used as marker proteins for subsequent classifications.

### Annotation of marker proteins

To map marker proteins into a three-dimensional space, we used the t-distributed stochastic neighborhood embedding (t-SNE) implemented in the ‘rtsne’ (v0.15) in R (20). Two hyperparameters of the perplexity (estimating how many elements each cluster may have) of 50 and the theta (the speed/accuracy trade-off) of 0.5 were optimized. We clustered the t-SNE coordinates of marker proteins (*n* = 1481) using mClust (v5.4.6), which assigned proteins to different clusters with probability based on the expectation-maximization algorithm on a mixture of Gaussians model (21). A total of 18 clusters were generated according to Bayesian Information Criteria (Supplementary Figure S2D). The identities of marker proteins and their cluster membership were available in Supplementary Table S1.

**Figure 2.**
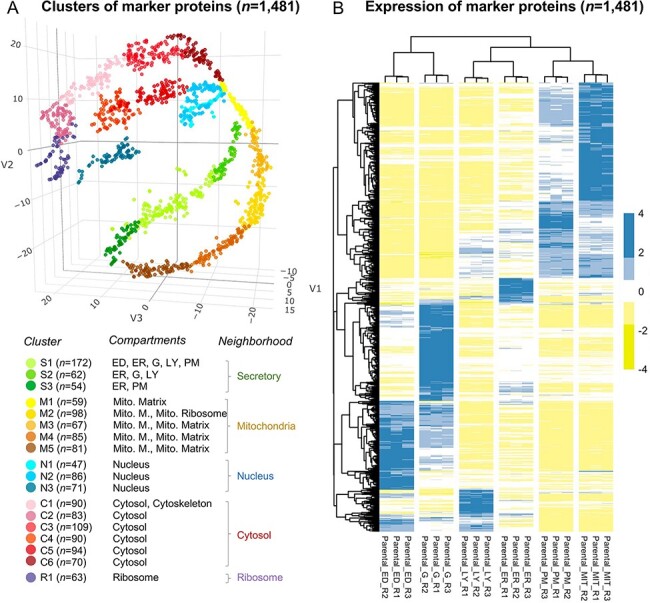
The annotation of the selected 1481 marker proteins. (A) A three-dimensional visualization for 18 clusters of marker proteins. Different colors represent clusters or neighborhoods. The number of marker proteins classified in each cluster was shown in brackets. Annotation for the subcellular compartments/organelles and their corresponding neighborhoods was present. ED, endosome; ER, endoplasmic reticulum, G, golgi apparatus; LY, lysosome; PM, plasma membrane; Mito, mitochondria; Mito. M., mitochondria membrane. (B) Differential expression of the selected marker proteins in organelles. R, replicate.

 We performed two rounds of annotation on the clusters of marker proteins. First, we used proteins with a unique subcellular location from UniProt, Gene Ontology, and an optimal marker set from mouse (22) according to the following five strategies: (i) proteins annotated exclusively in UniProt; (ii) in Gene Ontology; (iii) in either UniProt or Gene Ontology; (iv) in the intersection between UniProt and Gene Ontology and (v) in the intersection of ‘the union of UniProt and GO’ and ‘the marker protein set of the mouse’. Eleven subcellular compartments/organelles were annotated, including cytoskeleton, cytosol, nucleus, endosome, endoplasmatic reticulum, Golgi apparatus, lysosome, plasma membrane, mitochondrion, peroxisome and the ribosome. The fold change was calculated as ${\rm{FC}} = \left( {{\rm{b}}/{\rm{n}}} \right)/\left( {{\rm{B}}/{\rm{N}}} \right)$, where *b* is the protein number of the target organelle in the cluster, *n* is the total number of proteins in the cluster, *B* is the total protein number of the target organelle and *N* is the total number of proteins in all clusters. Significance levels were estimated using the hypergeometric test (‘Phyper’ in R) and were corrected for multiple testing (Benjamini–Hochberg) (Supplementary Table S2). We assigned the subcellular compartment to a given cluster if the FC for the given cluster is $ \ge $2 and the significance level is $<$0.05. The subcellular protein localization was required to be consistent in three out of five strategies. We successfully assigned endoplasmatic reticulum, lysosome and plasma membrane (Cluster 1), endoplasmatic reticulum, Golgi apparatus and lysosome (Cluster 2), endoplasmatic reticulum (Cluster 3), mitochondria (Clusters 4–8), nucleus (Clusters 9–11), cytoskeleton and cytosol (Cluster 12), cytosol (Clusters 13–17) and ribosome (Cluster 18) (Supplementary Table S2). Second, the 18 clusters were further annotated by a comprehensive gene ontology (GO) based cellular component enrichment analysis (i.e. a target-background approach) in Gorilla (23). The analysis resulted in the enrichment of endosome and Golgi apparatus in Cluster 1, plasma membrane in Cluster 3, the mitochondrial matrix in Cluster 4, mitochondrial membrane and mitochondrial ribosome in Cluster 5 and mitochondrial membrane and mitochondrial matrix in Clusters 6–8 (Supplementary Table S2, and Figure 2A).

We followed the definition of neighborhoods as ‘secretory’ (Clusters 1–3), ‘mitochondria’ (Clusters 4–8), ‘nuclear’ (Clusters 9–11) and ‘cytosol’ (Clusters 12–17) according to the known relationship of subcellular compartments (8). In addition, we classified ‘ribosome’ into one cluster (Cluster 18) and designated it as the ‘ribosome’ neighborhood (Supplementary Table S1).

### Machine-learning-based classification of subcellular protein localization

We used the support vector machine (SVM) with a Gaussian radial basis function kernel to classify the identified proteins by inputting the relative quantification of marker proteins. To build a classifier, we randomly split the marker proteins into the training (*n* = 984) and testing (*n* = 497) sets. Ten-fold cross-validation was used to avoid over-fitting on training data. We searched the best-fit parameters of the cost (i.e. misclassification rate) from ${10^{ - 10}} - {10^{10}}$ and gamma (i.e. controlling the shape of the segmented hyperplane) from ${10^{ - 10}} - 10$ across all classifiers using the ‘turn.svm’ function. The corresponding models were then applied to both marker and non-marker proteins to predict protein localization and its probability by the ‘e1071’ package (v1.7.4) in R. Proteins were preliminarily assigned to the cluster with the highest probability.

We retained the protein with the cluster assignment consistent in any two from triplicate; otherwise, the protein was labeled as ‘unclassified’. The prediction probability for each protein was averaged. Next, we set thresholds for the classification probability of clusters and neighborhoods based on the performance in the testing set. A true positive was defined if a protein of interest was correctly classified, and a false positive if incorrectly classified. A true negative was defined if a protein of non-interest was correctly classified, and a false negative if incorrectly classified. Finally, we plotted the precision (${\rm{TP}}/\left( {{\rm{TP}} + {\rm{FP}}} \right)$) and the recall rate (${\rm{TP}}/\left( {{\rm{TP}} + {\rm{FN}}} \right)$) for the cutoff and defined individual thresholds for each cluster. We selected the threshold to enable the probability maximizing the recall when the precision rate reaches 0.9.

For the neighborhood, the probabilities of the corresponding clusters were summed, and proteins were preliminarily assigned to the neighborhood with the highest probability. The threshold enabled the probability of maximizing the recall when the precision rate reached 0.95.

Since the precision rate was <0.9 or 0.95 for Cluster 11 and the ‘nucleus’ neighborhood, we used the F1 value (${\rm{F}}1 = 2{\rm{*}}\left( {{\rm{precision\,*\,recall}}} \right)/\left( {{\rm{precision}} + {\rm{recall}}} \right)$) to set the threshold. A total of 30 proteins appeared to be inconsistent between the cluster and the neighborhood level (e.g. it is classified as Cluster 10 at the cluster level but not classified to the nucleus at the neighborhood level). In this case, we assigned these proteins as ‘unclassified’ at both the cluster and neighborhood levels. The output of the cluster and neighborhood classifications and individual thresholds were available in Supplementary Table S3.

### Subcellular component localization network

The localization network was constructed based on proteins with a single neighborhood classification (*n* = 3803). Proteins classified into a single cluster were displayed as corresponding clusters, and proteins without cluster classifications were designated as neighborhood classifications. The network was visualized in Cytoscape (v.4.0.1).

### Localization of the protein domain and complex

We first obtained proteins with signal peptides, transit peptides and transmembrane domains from UniProt. Then, we searched the Pfam database (Pfam-A.hmm.gz) for annotating other protein domains using ‘hmmscan’ in HMMER (v3.3.2) (24). Significantly enriched domains were identified by fold enrichments and hypergeometric test (corrected for multiple testing using the Benjamini–Hochberg method). We identified 36 enriched domains considerably using the log2 (fold change) cutoff of 2 and a *q*-value of 0.05 (Supplementary Table S5). Next, we compared the similarity of the protein sequences of 50–3000 AA in the five neighborhoods using a clustering and comparison program of CD-HIT (v4.6.7) (http://weizhong-lab.ucsd.edu/cd-hit/). We obtained the proteins with sequence similarity >40% and then extracted the domains for proteins in each cluster in the Pfam database. The domains enriched in each neighborhood and the number of occurrences of the domains are obtained, and domains that appear to be less than three times in each neighborhood were filtered.

A list of human core complexes was obtained from the Comprehensive Resource of Mammalian Protein Complexes (CORUM) database, which collected experimentally verified mammalian protein complexes (25). For each member in the protein complex, the Pearson correlation was calculated, and proteins with the correlation values <0.8 were prefiltered out. The remaining 269 full-coverage protein complexes that all members identified after filtration were retained for subsequent analysis. We plotted the protein–protein interaction networks between the protein complex members using the ‘networkD3’ package (v0.4) in R.

### PSL-LCCL portal

For visualization and access to the subcellular protein localization, we created the PSL-LCCL portal developed by the shiny framework for R, which is available at http://www.igenetics.org.cn/project/PSL-LCCL/. The database provided access to both the raw fractionation data and the prediction for subcellular protein localization.

## Results

### Quantification of proteins in six membrane-bound organelles

The framework for the present study is illustrated in Figure 1. Six membrane-bound organelles were separated and enriched individually, and their proteins were qualitatively and quantitatively measured by MS. Western blotting for the organelle-specific markers validated the enrichment of each organelle (Supplementary Figure S1A). A total of 4464 proteins in six organelles with a high overlapping ratio of 92% (*n* = 4097) in triplicate showed the robustness of the proposed method (Supplementary Figure S1B). Furthermore, both principal component analysis (Supplementary Figure S1C) and heat map (Supplementary Figure S1D) showed a clear resolution of six different clusters based on the isolated organelles, further supporting the reproducibility of the subcellular fractionation by isolating and enriching specific organelles.

Of note, non-targeted organelle proteins were identified in ‘unexpected’ organelles, partly due to that the individual separation of organelles does not result in entirely pure fractions but rather an enrichment (Supplementary Figure S2A), as well as a relatively higher sensitivity of MS, as previously reported (12, 13). Therefore, we combined the identified proteins from six organelles and implemented a machine-learning algorithm to predict the subcellular protein location.

### Classification of marker proteins and their localizations

The classification based on a machine-learning algorithm depends strongly on the available markers. However, there is no widely accepted canonical organelle marker set. In addition, subcellular protein localization is often cell type-specific and dependent on the physiological context, further complicating the selection (6). A previous study suggested that marker proteins specifically localized in organelles were required to be highly replicable and have robust fractionation profiles under different conditions (8). Accordingly, 1481 marker proteins were selected by a quantitatively powerful method (Supplementary Figure S2B and Supplementary Table S1).

The marker proteins were classified into 18 clusters (Figure 2A and Supplementary Figure S2D), which were further annotated as different subcellular compartments. The distribution of proteins in t-SNE space indicated an internal connection between clusters; therefore, several clusters were adjacent (e.g. Clusters 1–3, Clusters 4–8, Clusters 9–11 and Clusters 12–17). Based on the known relationship of subcellular compartments (8), we used the definition of ‘neighborhood’ to merge adjacent clusters, i.e. ‘secretory’ (Clusters 1–3), ‘mitochondria’ (Clusters 4–8), ‘nuclear’ (Clusters 9–11) and ‘cytosol’ (Clusters 12–17). Cluster 18 of ‘ribosome’ was defined as the ‘ribosome’ neighborhood (Supplementary Table S1). The distinct expression profiles for the marker proteins among different organelles (Figure 2B) indicated the reliability of the selected marker proteins.

### A cluster-based subcellular protein localization

**Figure 3.**
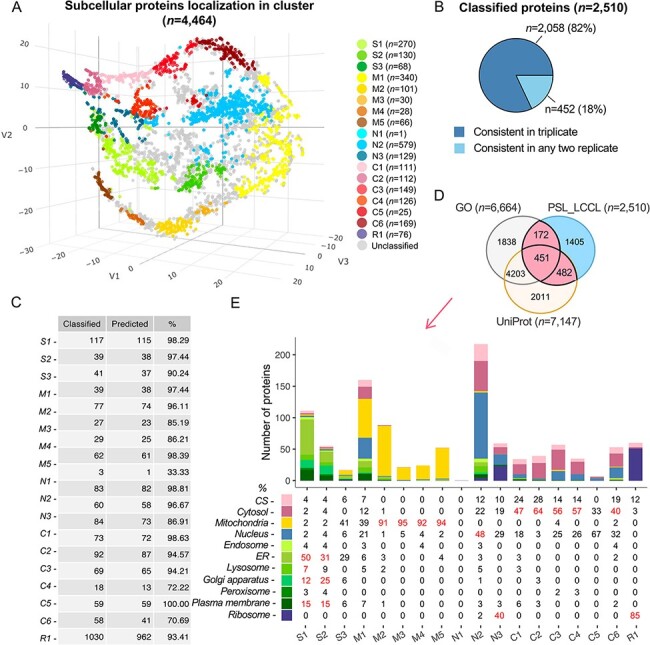
Subcellular localization of all identified proteins (*n* = 4464) at the cluster level. (A) The t-SNE classification for all proteins in a three-dimensional space. (B) The consistency of the classified proteins in replicate. (C) The classification accuracy of marker proteins at the cluster level. Classified: the number of marker proteins retained at each cluster level after filtering by threshold; predicted: the number of marker proteins correctly predicted. (D) The overlap of proteins classified with single cluster and proteins annotated with a single location in GO and UniProt. (E) Evaluation of single-cluster-classified proteins against proteins with single subcellular localization annotation from GO or UniProt.

For a rigorous assignment of all proteins into clusters, we used an SVM, a supervised machine-learning-based approach, to classify the proteins in each replicate (26). The marker proteins were divided into training (*n* = 984) and testing (*n* = 497) sets, balanced to cover the 18 clusters. Of the identified proteins in triplicate (*n* = 4464), we successfully classified 2510 (56%) proteins into clusters (Figure 3A), highly consistent in triplicate (Figure 3B). To improve the classification accuracy, we merged the classifications from triplicate and used the testing set marker proteins to set thresholds for each cluster. The prediction accuracy for marker proteins at the cluster levels was increased from 86.0 to 93.4% with the cutoff threshold for each cluster (Figure 3C and Supplementary Table S4; see Methods and Supplementary Table S3.1). The accuracy for cluster-based marker prediction indicated higher robustness for the overall prediction accuracy. We then compared the predicted single-localized proteins (*n* = 2510) with those having single localization in the UniProt (*n* = 7147) or GO (*n* = 6664) and noted that 44% of proteins (*n* = 1105) were consistent as single-localization proteins (Figure 3D). Enrichment analysis verified that subcellular components were correctly assigned at the cluster level (Figure 3E), e.g. S1 was annotated with ‘endoplasmic reticulum’, ‘lysosome’, ‘Golgi’ and ‘plasma membrane’, consistent with our classification.

### A neighborhood-based subcellular protein localization

**Figure 4.**
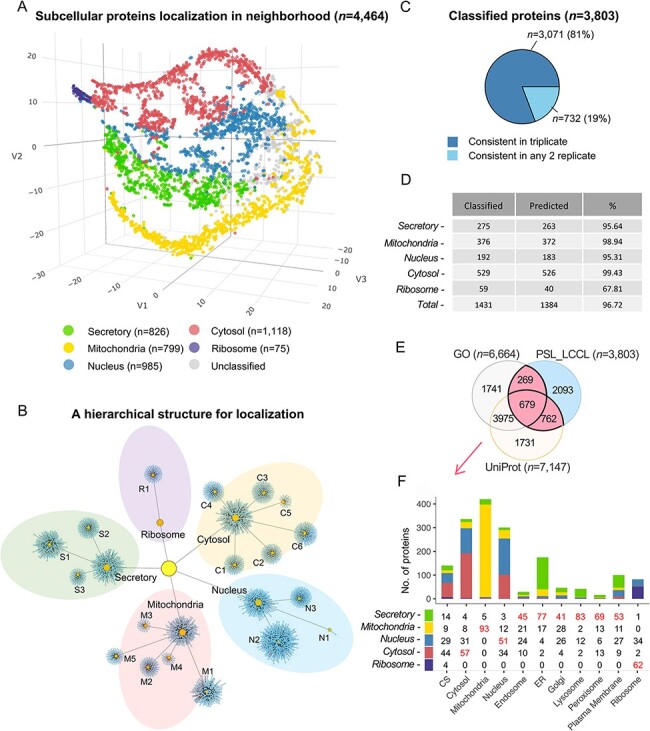
Subcellular localization of the identified proteins (*n* = 4464) at the neighborhood level. (A) A projection of all identified proteins in a three-dimensional space at the neighborhood level. (B) A hierarchical network of the classification at both cluster and neighborhood levels. (C) The consistency of the classified proteins in replicate. (D) The classification accuracy of marker proteins at the neighborhood level. Classified: the number of marker proteins retained at each neighborhood level after threshold filtering; predicted: number of marker proteins correctly predicted. (E) The overlap of single-neighborhood-classified proteins and proteins annotated with a single location in GO and UniProt. (F) Evaluation of single neighborhood classified against proteins with single subcellular localization annotation from GO or UniProt.

Due to the cluster relatedness and a limited coverage depth of proteins, only half (56%) were classified as the cluster level. We followed the definition of ‘neighborhoods’ (8) to classify proteins at a higher level according to the known relationship of subcellular compartments (Figure 4A), where neighborhoods were well distinguished. As a result, an increased proportion of 85% proteins (*n* = 3803) were classified into neighborhoods, i.e. approximately 1300 proteins were rescued at the neighborhood level, significantly greater than the coverage of 56% at the cluster level. A localized network demonstrated an overview of the classification by mapping proteins into specific clusters or neighborhoods, as well as their relationship (Figure 4B). The neighborhood-based classification had obtained high consistency in triplicate (Figure 4C). The classification accuracy indicated by marker proteins was increased from 95.2% to 96.7% by a cutoff threshold for each neighborhood, respectively (Figure 4D and Supplementary Table S4). We compared the proteins classified in a single location annotated in the neighborhood (*n* = 3803) with proteins having single localization in UniProt or GO, and 45% (*n* = 1710) proteins were consistent (Figure 4E). Organelle was also correctly enriched at the neighborhood level (Figure 4F), e.g. the ‘secretory’ neighborhood was annotated to include ‘endosome’, ‘endoplasmic reticulum’, ‘lysosome’, ‘Golgi’, ‘plasma membrane’ and ‘peroxisome,’ consistent with our classification.

### Comparison of subcellular protein localization with public resources

**Figure 5.**
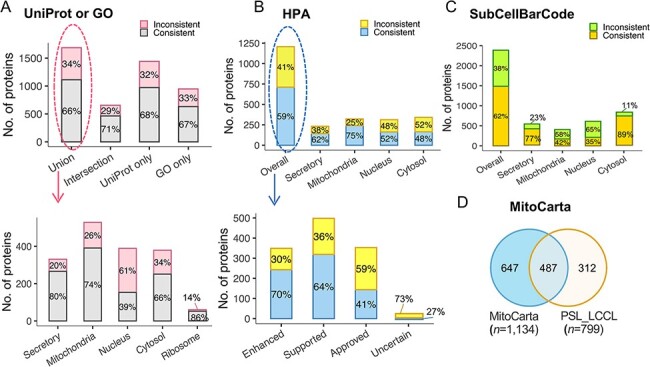
The evaluation of subcellular protein localization in PSL-LCCL against the public databases. (A) An agreement of neighborhood-based classification of proteins in PSL-LCC with proteins having unique localization annotated in UniProt or GO (upper). An agreement between PSL-LCC classifications and UniProt and GO single-location proteins of each neighborhood (below). Union, the union of UniProt and GO; Intersection, the overlap proteins in UniProt and GO. (B) An agreement of the neighborhood-based classifications in PSL-LCCL and proteins having unique localization annotated in Human Cell Atlas (upper) and their corresponding reliability score (below). (C) An agreement of the neighborhood-based classification in PSL-LCCL and proteins localized in SubCellBarCode Orre *et al.* (8). (D) An agreement of proteins localized in mitochondria in PSL-LCCL with MitoCarta Rath *et al.* (28).

To further evaluate our classification, we assessed proteins classified into a single neighborhood (*n* = 3803) against single-localization proteins annotated in the public database. The protein localization of the public database was first assigned to the five defined neighborhoods. Of the 1686 proteins overlapped with UniProt and GO, an agreement was increased from 65.9% to 70.6% for proteins with the same localization annotated in both UniProt and GO (Figure 5A). We also compared the classification with that assigned a single subcellular location in the Cell Atlas (27) (*n* = 4647) (Figure 5B). The overall agreement was relatively lower (59.0%) than GO or UniProt (65.9%). However, the consistency was associated with the reliability score, e.g. an agreement of 70% for proteins with the highest score of ‘Enhanced’ (*n* = 243), 64% with ‘Supported’ (*n* = 319), 41% with ‘Approved’ (*n* = 144) and 27% with ‘Uncertain’ (Figure 5B). The overall concordance with SubCellBarCode (8) was 62% (Figure 5C). In the ‘Cytosol’ neighborhood, there was a consistent rate of 89%. It should be noted that inconsistency may be due to the ‘ribosome’ being classified into the ‘nuclear’ neighborhood in SubCellBarCode. Of the 799 proteins localized in mitochondria, 61% (*n* = 487) were present in the mitochondrial database (28) (MitoCarta 3.0) (Figure 5D). In summary, our external validation of the classifications confirmed the classified subcellular protein localization.

### Subcellular distribution of protein domains and complex

**Figure 6.**
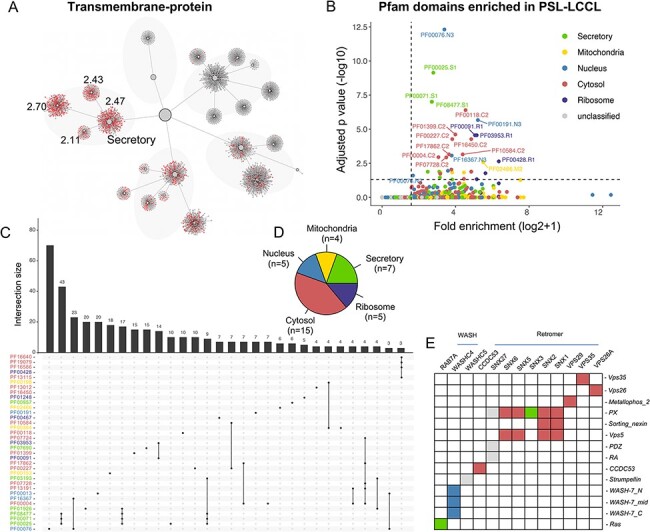
The effects of domains on subcellular proteins localization. (A) The localization of proteins with transmembrane domain in the hierarchical structure. Enriched locations (*P* < 0.05) are indicated. (B). Enrichment analysis for Pfam domains in our neighborhoods. The cutoff for fold enrichment was two (*adjusted *P*-value* < 0.05). (C). Protein domains significantly enriched in PSL-LCCL. (D) The number of protein domains that are significantly enriched in neighborhoods. (E) The domain of the retromer complex.

Protein domains may play a role in subcellular protein localization, e.g. a protein with a transit peptide can be transported to the mitochondria (29), and a signal peptide targets the protein to the ‘secretory’ neighborhood (8). The mapping of proteins with various domains (e.g. transmembrane, signal and transit) into the localization network showed that proteins with different domains were enriched in expected neighborhoods (Figure 6A and Supplementary Figure S3). The association between protein domains and their localizations showed that 36 domain families annotated in Pfam were significantly enriched in specific clusters or neighborhoods (*q*  $<$ 0.05) (Figure 6B, C and Supplementary Table S5). Of note, the cytosol demonstrated the most enriched domains, consistent with its function as a protein storage reservoir (Figure 6D).

We also clustered proteins in each neighborhood using CD-HIT. For protein clusters with sequence similarity >40%, 27 domains appeared to more than three times in secretory, 2 in mitochondria, 10 in nucleus, 22 in cytosol and 2 in ribosome (Supplementary Table S5). We showed that protein with AAA, AAA_lid_3 or Prot_ATP_ID_OB domains were more likely to be localized in cytosol and proteins with Arf domain might be localized in secretory. In addition, RRM, KH_1 or Annexin domains might play a role in nucleic transportation and Tublin or Tublic_C domains were involved in ribosome transportation.

We next investigated the domains and their localization of the members in the retromer complex, which comprises a VPS26-VPS29-VPS35 heterotrimer implicated in cargo recognition and various combinations of sorting nexin (SNX) proteins, contributing to membrane recruitment and formation of recycling tubules (30). The SNX protein family has different domains (Figure 6E), binding with VPS35-VPS26-VPS29 trimer to mediating distinct endosomal trafficking pathways. We showed that members in the retromer complex and its associated proteins were primarily located in the cytosol (Figure 6E). Of the domains of cargo protein for the retromer complex, $>$300 membrane proteins depending on retromer for their localization at the cell surface were identified (31). In addition, the annotated ‘MFS_1’ and ‘Mito_carr’ domain was significantly enriched in the ‘secretory’ and ‘mitochondria’ neighborhood, respectively.

**Figure 7.**
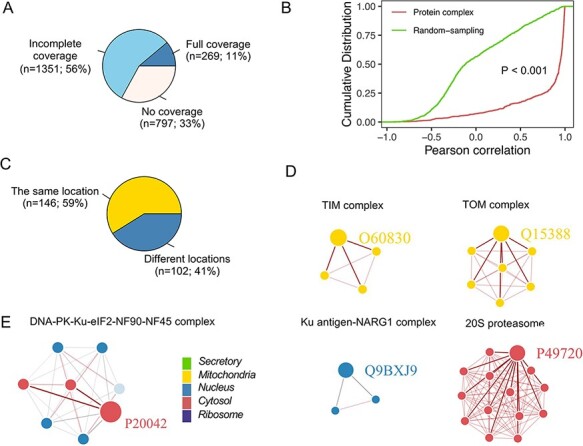
Subcellular localization for the protein complex. (A) The coverage of CORUM complexes in PSL-LCCL. CORUM: the comprehensive resource of mammalian protein complexes; full: all the protein complex members were present in our data; no coverage: members in the protein complex were not identified in our study. (B) A cumulative plot of the correlation for the protein complex members in CORUM. Random sampling was from a random sampling of correlations for nonprotein complex members. (C) The consistency of the localization of the protein complex members. (D) Examples of members of protein complexes located in the same neighborhood. (E) Examples of members of protein complexes located in different neighborhoods.

Protein complexes composed of multiple proteins play critical roles in various biological processes (32). Using the CORUM database, we assessed the colocalization of members from protein complex and their correlation (25) (Figure 7A), which was significantly greater than that in a random sampling of nonprotein complex members (Figure 7B). Furthermore, proteins in some complexes located in the same neighborhood exhibited high interactions with each other (Figure 7C). For example, proteins in the TOM and TIM complex, localized in the outer and inner mitochondrial membranes, respectively (33), showed apparent colocalization (the correlation between any two members was $>$0.8) (Figure 7D). However, not all proteins in complexes were located in the same neighborhood (Figure 7C), e.g. five members (Q12906, P13010, P78527, Q12905 and P12956) in the DNA-PK-Ku-eIF2-NF90-NF45 complex are localized in the nucleus (Figure 7E). In comparison, three members (P41091, P05198 and P20042) were classified into the cytosol, poorly correlated with other members. One possible explanation was that proteins in the nucleus are related to DNA double-strand break repair (34), and proteins in the cytoplasm are the subunits of the Eukaryotic translation initiation factor 2, involved in the early steps of protein synthesis.

### A database of protein subcellular location

We created a database of PSL-LCCL, including two layers of information. At the protein level, it included the classification probabilities of proteins in the 18 clusters and 5 neighborhoods. For demonstration purpose, we created a simple cell map to visualize the localization of a given protein by keyword query. Users can also view the proteins of interest in the organelles. When multiple proteins are inquired, the classification for all proteins will be displayed simultaneously, enabling a convenient way to compare the localization of various proteins. Examples of well-known compartment markers were shown (Supplementary Figure S4). At the complex level, our resources provided information on the location of members in the protein complex and their interaction from the CORUM database (25). Users can view the protein complex of interest by inquiring about the name of the complex or the included members (e.g. Fig. 7D, E). The database is accessible via a web interface (http://www.igenetics.org.cn/project/PSL-LCCL/).

## Discussion

We isolated and enriched six cytosolic membrane-bound organelles individually and implemented a machine-learning-based algorithm. We predicted the subcellular protein localization for the identified proteins in the SK_HEP1 cell line. We successfully classified 2510 (56%) and 3803 (85%) out of 4464 identified proteins into 18 clusters and 5 neighborhoods, respectively. The prediction accuracy for marker proteins and the comparison of the subcellular protein localization with the well-known public resources confirmed the reliability of our results. Furthermore, the classification enabled investigating the association between subcellular localization with protein domains and complexes. For resource convenience, a user-friendly subcellular proteome database of ‘PSL-LCCL’ for SK_HEP1 was provided.

Recently, MS and machine-learning algorithms have been used to study subcellular protein localization (5, 6, 8, 35). The machine-learning-based algorithm is a ‘boundary’ method, and thus marker proteins near the edge of clusters are essential for distinguishing clusters (6). Since there lack of canonical organelle-specific marker proteins, compiling a suitable set of marker proteins played a vital role for downstream analysis. A recent meta-analysis demonstrated the benefits of combining various data sources for selecting markers (22). We therefore compiled a list of marker proteins by incorporating unbiased annotations of single-localization proteins from different sources (22), and the prediction errors based on a single annotation could be reduced. For example, when single-localization proteins were annotated in either GO or UniProt, Cluster 15 was classified into ‘nucleus’ or ‘cytosol,’ respectively; however, it was correctly classified as ‘cytosol’ when single-localization proteins were annotated from both GO and UniProt. Marker proteins were selected based on the principle that proteins with high reproducibility and robustness under different conditions can be used as marker proteins for subcellular localization [8]. Here, we used the protein stably expressed in the parental and VPS35-KO cells as the marker proteins, strengthening the application of the selected marker proteins.

The annotation for each cluster (i.e. the assignment of organelle based on the public resources) may not be unique (Figure 2). Organelles may share similar components since proteins are frequently transported between cytosolic organelles (6); e.g. lysosomes are reformed from endolysosomes. Since distinguishing these organelles remains a challenge, we merged them as the ‘secretory’ neighborhood. The number of successfully classified proteins increased from 56% (cluster-based) to 85% (neighborhood-based) (Figures 3A and 4A). In addition, the classification in triplicate obtained high consistency at both the cluster and neighborhood levels (Figures 3B and 4C). Different technologies and statistical approaches may also result in different subcellular protein localizations (1), thus evaluating the prediction accuracy remains difficult. Usually, the evaluation of the subcellular protein localization was to compare that annotated in protein databases (e.g. UniProt, GO and HPA) (27) as well as single-organelle proteome databases. The external verification also confirmed the robustness of our subcellular protein localization (Figure 5).

Previous studies have shown that protein domains have implications in the localization of proteins, e.g. signal peptide (8) and transit peptide (29). Of the 36 protein domains that were significantly enriched (Figure 6B), 9 (out of 15 identified) were consistent with the classified subcellular localization in SubCellBarCode (8). These findings suggested an association between protein domain and subcellular localization. Protein crystallography revealed that protein domain as the fundamental unit of protein may have strong combinatorial capabilities to form new proteins (36). Our findings also showed a greater correlation between the members of the protein complexes than that between noncomplex members (Figure 7B), suggesting a prominent colocalization among the complex members (8, 37).

The present study has several limitations. First, proteins in ‘unexpected’ organelles were identified under the proposed method. An impure subcellular fractionation for six membrane-bound cytosolic organelles may include proteins in unseparated cytosolic or nucleic components. A consistent rate of 89% cytosolic proteins classified in the present study and SubCellBarCode was obtained. However, the lack of separation for cytosolic and nucleic components resulted in a relatively lower protein coverage. Separating more organelles could improve the classification accuracy and the coverage depth. Second, although our method can determine the single dominant localization for proteins, proteins with multilocalization remain undetermined. A previous study showed that multilocalization of proteins was uncommon, and $<$10% of proteins were multilocalized (8). Although the Human Cell Atlas reported that >50% of proteins were localized in multiple subcellular locations (27), these proteins were associated with low-reliability scores. Moreover, some proteins may be incorrectly classified into multiple compartments due to different qualities and sensitivities imposed by various methods. Further study of multilocalized proteins needs more attention. Third, an experimental validation *in vitro* could improve the reliability of the localization, especially for proteins with inconsistent annotations. However, it may be beyond the scope of the present study. Finally, protein relocation is essential for cell signal transmission and rapid adaptation to environmental changes. A dynamic organelle map can be used to identify global translocation proteins for our further study.

In conclusion, our study characterized subcellular protein localization of proteins identified in six membrane-bound cytosolic organelles for the SK_HEP1 cell line. A protein complex interaction map integrating member localization and protein–protein interactions was provided. Our resources have implications for further research on the proteomics of liver cancers.

## Supplementary Material

baab087_Supp
