# Supplementary material for: PSL-LCCL: a resource for subcellular protein localization in liver cancer cell line SK_HEP1
Source: Database (Oxford). 2022 Jan 17;2022:baab087. doi: 10.1093/database/baab087 (PMC9248857; doi:10.1093/database/baab087)
Supplement: baab087_Supp [file baab087_supp.zip › supplementary.docx]

PSL-LCCL: A Resource for Subcellular Protein Localization in Liver Cancer Cell Line SK-hep1

Fang Huang^2,*^, Xia Tang^1,*^, Bo Ye^2^, Songfeng Wu^3,#^, and Keyue Ding^1,#^

1 Medical Genetic Institute of Henan Province, Henan Provincial People’s Hospital, Henan Key Laboratory of Genetic Disease and Functional Genomics, National Health Commission Key Laboratory of Birth Defect Prevention, Henan Provincial People’s Hospital of Henan University, People’s Hospital of Zhengzhou University, Zhengzhou, Henan Province, 450003, People’s Republic of China

2 Department of Bioinformatics, School of Basic Medicine, Chongqing Medical University, Chongqing, 400016, People’s Republic of China

3 State Key Laboratory of Proteomics, Beijing Proteome Research Center, National Center for Protein Sciences (Beijing), Research Unit of Proteomics & Research and Development of New Drug of Chinese Academy of Medical Sciences, Institute of Lifeomics, Beijing 102206, People’s Republic of China

*, Contribute equally

#, Correspondence: Songfeng Wu, Ph.D., or Keyue Ding, Ph.D.

Email: [songfengwu@126.com](mailto:songfengwu@126.com), or [ding.keyue@igenetics.org.cn](mailto:ding.keyue@igenetics.org.cn)

#### **Supplementary tables (in Excel tables)**

Table S1. A compiled list of marker proteins in the present study

Table S2.1. The primary and secondary annotation for marker proteins

Table S2.2. A secondary annotation for marker proteins

Table S3.1. The classification threshold used for clusters and neighborhoods

Table S3.2. The classification probability and results at clusters and neighborhoods

Table S4.1. The classification accuracy of marker proteins at the cluster level

Table S4.2. The classification accuracy of marker proteins at the neighborhood level

Table S5.1. An analysis of domain enrichment

Table S5.2. An enrichment analysis for Pfam protein domains

Table S5.3 CD-HIT analysis for protein domains

Table S6. A list of antibodies used for Western blot

#### **Supplementary figures**

Fig S1. Quality control. (A). Western blotting for protein markers against the organelle. KO, *VPS35*-Knockout. ER, endoplasmic reticulum; PM, plasma membrane; LY, lysosome; and Mito, mitochondria; (B). The overlap of proteins identified in triplicate; (C). Principle component analysis for proteins identified in six membrane-bound organelles in triplicate; and (D). A heat map of the quantified proteins by mass spectrometry from six membrane-bound organelles showed that each organelle has a specific protein expression profile.

Fig S2. Marker selection and classification. (A). A heat map showed that the isolated organelle may contain proteins from other organelles; (B). A procedure for selecting marker proteins. PCC: Pearson correlation coefficient; min: minimal; (C). The correlation between the selected marker proteins in both the parental and *VPS35*-KO SK-Hep1. The marker proteins were expressed in both cells; and (D). The determination of the cluster number by the Bayesian Information Criteria (BIC). The red line showed the number of clusters used (*n*=18).

Fig S3. Localization of proteins with signal or transit peptide based on localization network. Enriched locations (*p* < 0.05) are indicated.

Fig S4. Examples for well-known compartment markers. (A). Global positioning map for five well-known compartment markers; (B). The classification probability at the cluster level for five well-known compartment markers; and (C). The classification probability at the neighborhood level for five well-known compartment markers.
